# Endothelial-derived extracellular microRNA-92a promotes arterial stiffness by regulating phenotype changes of vascular smooth muscle cells

**DOI:** 10.1038/s41598-021-04341-1

**Published:** 2022-01-10

**Authors:** Chen Wang, Haoyu Wu, Yuanming Xing, Yulan Ye, Fangzhou He, Qian Yin, Yujin Li, Fenqing Shang, John Y.-J. Shyy, Zu-Yi Yuan

**Affiliations:** 1 Department of Cardiology, First Affiliated Hospital of Xi’an Jiaotong University, Xi’an, China; 2 Translational Medicine Centre, Xi’an Chest Hospital, Xi’an, China; 3 Cardiovascular Research Center, School of Basic Medical Sciences, Xi’an Jiaotong University Health Science Center, Xi’an, China; 4 Division of Cardiology, Department of Medicine, University of California, La Jolla, San Diego, CA USA; 5 Department of Cardiology, Xi’an GaoXin Hospital, Xi’an, China; 6 Department of Life Sciences and Medicine, Northwestern University, Xi’an, China

**Keywords:** Cell biology, Biomarkers, Diseases, Medical research

## Abstract

Endothelial dysfunction and vascular smooth muscle cell (VSMC) plasticity are critically involved in the pathogenesis of hypertension and arterial stiffness. MicroRNAs can mediate the cellular communication between vascular endothelial cells (ECs) and neighboring cells. Here, we investigated the role of endothelial-derived extracellular microRNA-92a (miR-92a) in promoting arterial stiffness by regulating EC–VSMC communication. Serum miR-92a level was higher in hypertensive patients than controls. Circulating miR-92a level was positively correlated with pulse wave velocity (PWV), systolic blood pressure (SBP), diastolic blood pressure (DBP), and serum endothelin-1 (ET-1) level, but inversely with serum nitric oxide (NO) level. In vitro, angiotensin II (Ang II)-increased miR-92a level in ECs mediated a contractile-to-synthetic phenotype change of co-cultured VSMCs. In Ang II-infused mice, locked nucleic acid-modified antisense miR-92a (LNA-miR-92a) ameliorated PWV, SBP, DBP, and impaired vasodilation induced by Ang II. LNA-miR-92a administration also reversed the increased levels of proliferative genes and decreased levels of contractile genes induced by Ang II in mouse aortas. Circulating serum miR-92a level and PWV were correlated in these mice. These findings indicate that EC miR-92a may be transported to VSMCs via extracellular vesicles to regulate phenotype changes of VSMCs, leading to arterial stiffness.

## Introduction

Arterial stiffness is considered a complication of hypertension and a comorbidity of various cardiovascular diseases^1,2^. Clinically, arterial stiffness can be assessed non-invasively by measuring brachial-ankle pulse wave velocity (PWV). Dysfunctional endothelium plays a major role in the pathology of vascular stiffness^3^. Vascular smooth muscle cell (VSMC) plasticity, characterized by a dedifferentiated transition from a contractile to a proliferative phenotype, also contributes to arterial stiffness^4^. However, physiological communication between vascular endothelial cells (ECs) and VSMCs is an integral part of vascular homeostasis^5,6^. For example, nitric oxide (NO), synthesized and released by ECs, confers VSMC relaxation and maintains the contractile phenotype of VSMCs^7^.

MicroRNAs (miRNAs) are small non-coding RNAs. By targeting the 3′-untranslated region (3′-UTR) of the targeted mRNA, miRNAs epigenetically regulate gene expression in almost all biological systems. Increasing evidence indicates that extracellular miRNAs can serve as biomarkers for the diagnosis, treatment, and prognosis of cardiovascular diseases^8–10^. In addition, miRNAs can function as second messengers mediating cellular communication between ECs and neighboring cells within the vascular wall^11–13^. Elevated miR-92a level in the cardiovascular system is linked to the pathophysiology of cardiovascular diseases^14–16^. Circulating miR-92a level was found correlated with clinical markers of hypertension such as 24-h ambulatory blood pressure parameters, carotid intima-media thickness, and carotid-femoral PWV, so miR-92a has an important role in the pathogenesis of hypertension^16^.

We previously showed that increased miR-92a level causes EC dysfunction by targeting genes such as Krüppel-like factor 2 (KLF2), KLF4, and sirtuin 1 (SIRT1), crucial for a homeostatic endothelium^14^. EC miR-92a can be transported to macrophages via extracellular vesicles (EVs) to downregulate KLF4 levels^11^. Yet, it is not known whether dysregulated miR-92a level in ECs during hypertension onset contributes to arterial stiffness and if so, whether the underlying mechanism involves EC–VSMC communication via miR-92a.

Hence, the objective of this study was to investigate the role of miR-92a in the EC–VSMC crosstalk and its consequential effect in arterial stiffness. Our results reveal an inverse correlation between circulating miR-92a level and PWV in humans and mice. The underlying mechanism involves increased miR-92a level in ECs modulating VSMC plasticity. Locked nucleic acid-modified antisense miR-92a (LNA-miR-92a) ameliorated angiotensin II (Ang II)-induced hypertension in mice, which suggests a therapeutic potential of miR-92a antagonists in ameliorating arterial stiffness.

## Results

### Increased serum miR-92a level in patients with hypertension

**Figure 1:**
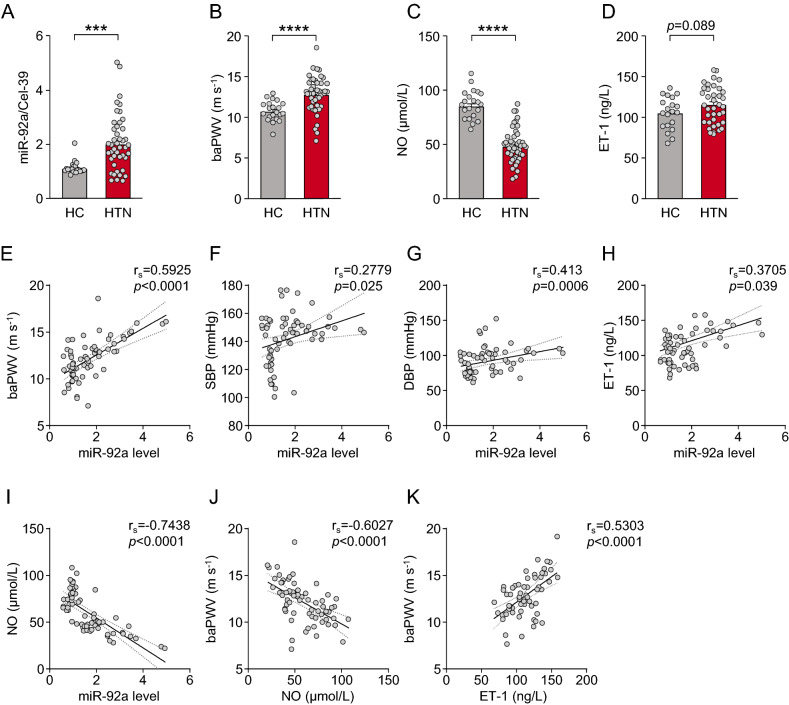
Increased serum miR-92a level in patients with hypertension. (**A**) qPCR analysis of serum miR-92a level in patients with hypertension (HTN) (n = 44) and healthy controls (HC) (n = 21). Cel-miR-39 was used as a spike-in control. The data are fold change normalized to the averaged level of HCs. (**B**) Brachial–ankle pulse wave velocity (baPWV) of patients and controls. (**C**, **D**) Serum NO and ET-1 levels. (**E**–**I**) Correlations between serum miR-92a level and baPWV (**E**), SBP (**F**), DBP (**G**) serum ET-1 (**H**) and NO (I). (**J**, **K**) Correlation between baPWV and serum NO (**J**) and ET-1 (**K**) levels. Data are mean ± SEM. Normally distributed data were analyzed by the two-tailed Student *t* test (**C**, **D**), and the two-tailed Student *t* test with Welch correction (**B**) between 2 indicated groups. Non-normally distributed data were analyzed by the Mann–Whitney U test (**A**) between 2 indicated groups. *p < 0.05, **p < 0.01, ***p < 0.001, ****p < 0.001. *r*_*s*_ Spearman correlation coefficient.

We collected blood from two groups of donors: the patient group (n = 44) with a first diagnosis of hypertension and heathy controls (n = 21) (patient demographics in Table 1). Serum was isolated from the blood and level of miR-92a was measured. As compared with healthy controls, hypertensive patients showed significantly higher circulating miR-92a level (Fig. 1A). Additionally, BMI and levels of blood glucose and triglycerides were higher in hypertensive patients than controls (Table 1). To investigate whether circulating levels of miR-92a were correlated with arterial stiffness, we compared the two groups for PWV, an indicator of arterial stiffness. PWV was higher in hypertensive patients than controls (Fig. 1B). We and others have previously shown that circulating miR-92a level is inversely correlated with endothelial nitric oxide synthase (eNOS)-derived NO bioavailability^14,19^. As anticipated, serum NO level was significantly lower in hypertensive patients than controls (Fig. 1C), but serum ET-1 level was higher (Fig. 1D). Serum miR-92a level was positively correlated with systolic and diastolic blood pressure (SBP and DBP), brachial-ankle PWV (baPWV), and serum ET-1 level (Fig. 1E–H) but inversely correlated with serum NO level (Fig. 1I). Furthermore, baPWV was inversely correlated with serum NO level but positively with serum ET-1 level (Fig. 1J,K). These results suggest that circulating miR-92a level was indicative of endothelial dysfunction and arterial stiffness during hypertension onset.

**Table 1:** Baseline characteristics of healthy controls and patients with hypertension.

	Controls (n = 21)	Hypertension patients (n = 44)	*p* value
Age (years)	41.05 ± 1.81	45.34 ± 1.48	0.09
Sex (male)	8 (38%)	27 (61.4%)	0.0784
Current smoker	6 (28.57%)	13 (29.55%)	0.936
SBP (mmHg)	124.4 ± 2.44	151.6 ± 1.41	< 0.0001
DBP (mmHg)	75.38 ± 1.59	99.09 ± 2.69	< 0.0001
BMI (kg/m^2^)	22.76 ± 0.84	26.04 ± 0.66	0.0045
Total cholesterol (mmol/L)	4.84 ± 0.17	5.02 ± 0.13	0.41
Triglycerides (mmol/L)	1.22 ± 0.16	2.02 ± 0.17	0.001
LDL-C (mmol/L)	2.33 ± 0.12	2.48 ± 0.07	0.26
Blood glucose (mmol/L)	5.07 ± 0.13	5.98 ± 0.21	0.0002

Data are mean ± SD or number (%).

*SBP* systolic blood pressure, *DBP* diastolic blood pressure, *BMI* body mass index, *TC* total cholesterol, *TG* triglycerides, *LDL* low-density lipoprotein.

### Ang II induces miR-92a and EC dysfunction

**Figure 2:**
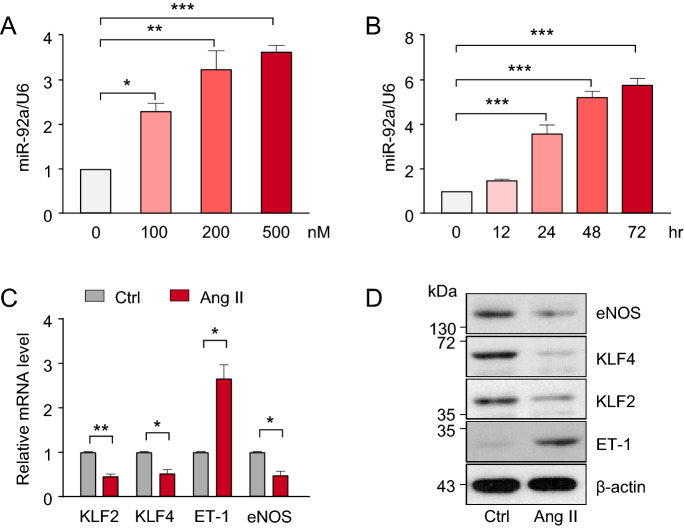
Ang II induces miR-92a and EC dysfunction. (**A**) miRNA qPCR analysis of miR-92a level in HUVECs treated with various concentrations of Ang II for 24 h. (**B**) Relative miR-92a level in HUVECs treated with Ang II (200 nM) for different times. (**C**, **D**) HUVECs were treated with Ang II (200 nM) or PBS (Ctrl) for 24 h. mRNA and protein levels of KLF2, KLF4, eNOS, and ET-1 were measured by qPCR and western blot, respectively. Data are mean ± SEM from 3 independent experiments in (**A**–**C**). Parametric data were analyzed by the one-way ANOVA between multiple groups (**A**, **B**) and by the two-tailed Student-t test with Welch correction between 2 indicated groups (**C**). *p < 0.05, **p < 0.01, ***p < 0.001. The eNOS, KLF4, KLF2, ET-1 and β-actin bands were cropped from full gels (Supplementary Fig. S1a).

Elevated Ang II level during the hypertension onset has a detrimental role in the cardiovascular system. In vitro, Ang II treatment often causes EC dysfunction. Thus, we examined whether miR-92a in ECs could be induced by Ang II. Ang II treatment increased miR-92a level in ECs dose- and time-dependently (Fig. 2A,B). Genes crucial for homeostatic ECs, including eNOS, KLF2, and KLF4, are miR-92a targets^14^. Ang II treatment decreased the mRNA and protein levels of eNOS, KLF2, and KLF4 in ECs (Fig. 2C,D), but the expression of pro-inflammatory and -fibrotic ET-1 was increased (Fig. 2C,D). Together, these results indicate that Ang II-induced EC dysfunction might be mediated by elevated miR-92a level.

### miR-92a regulates phenotype changes of VSMCs

**Figure 3:**
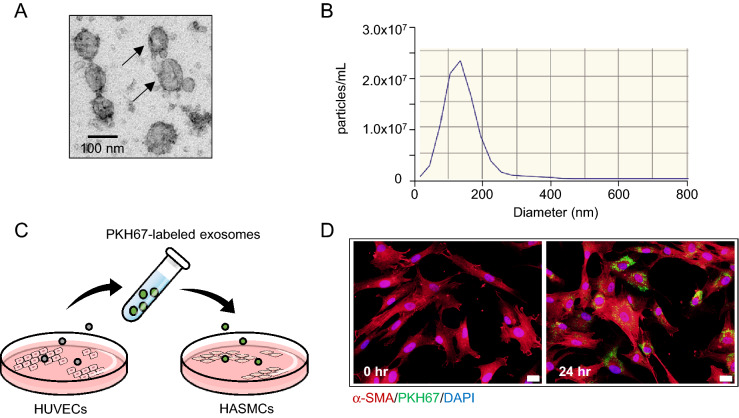
HUVEC-derived exosomes are transported to HASMCs. (**A**) Electron microscopy image of HUVEC-derived exosomes, showing a size of approximately 100 nm in diameter. Scale bar: 100 nm. (**B**) Size distribution of HUVEC-derived exosomes was detected by Nanoparticle Tracking Analysis (NTA). (**C**, **D**) Exosome uptake experiment. HUVEC-derived exosomes were labeled with green fluorescent dye (PKH67) and incubated with HASMC. HASMCs were incubated with PKH67-labeled exosomes from HUVECs for 24 h and fixed for confocal imaging. HASMC were stained with α-SMA (red) and DAPI (blue). Scale bar: 20 μm. The schematic diagram (**C**) was generated by Microsoft PowerPoint & Visio 2019 MSO (16.0.14326.20384), 64 bits, http://www.microsoft.com.

Because the contractile-to-synthetic phenotype change of VSMCs contributes to arterial stiffness^4^, we explored whether increased miR-92a level in ECs could affect the VSMC phenotype. Chang et al. have shown that miR-92a can be secreted from endothelial cells into exosomes to regulate macrophage function^11^. First, the exosomes were isolated from the conditioned media of human umbilical vein ECs (HUVECs) and confirmed by electron microscope and nanoparticle tracking analysis (NTA) with a typical size of approximately 100 nm and a characteristic cup-shaped morphology (Fig. 3A,B). To study whether EC-derived exosomes transfer to VSMCs, we used PKH67 to label the secreted exosomes and cultured VSMCs with the labeled exosomes. The exosomes uptake experiment showed that VSMCs uptake HUVEC-derived exosomes (Fig. 3C,D).

**Figure 4:**
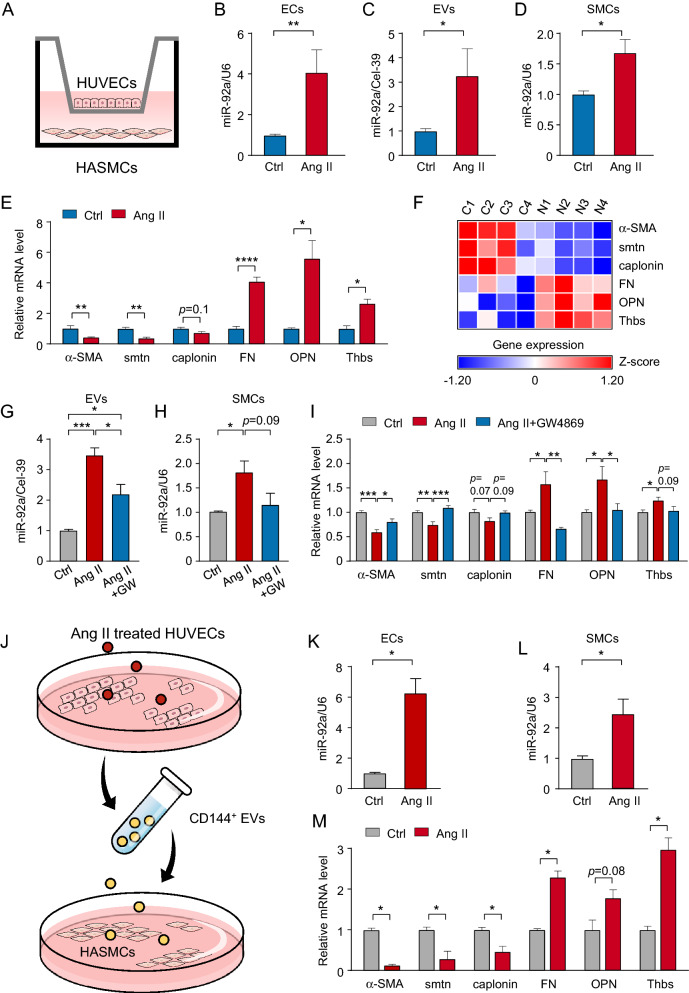
miR-92a regulates phenotype changes of VSMCs. (**A**) In a static coculture system, HUVECs were treated with 200 nM Ang II or PBS (Ctrl) for 24 h before co-culture with VSMCs. (**B**–**D**) Extracellular vesicles (EVs) were isolated from conditioned media of the Ang II- or PBS-treated HUVECs. qPCR analysis of miR-92a level in HUVECs (**B**), EVs (**C**) and VSMCs (**D**). (**E**) The relative expression of contractile genes (α-SMA, smoothelin [smtn] and calponin) and proliferative (fibronectin [FN], osteopontin [OPN] and thrombospondin [Thbs]) genes in VSMCs co-cultured with Ang II- or PBS-treated HUVECs. (**F**) Heatmap shows the expression of differentially expressed genes according to Z-scores in carotid arteries with intima injury (N1-4) and controls (C1-4). (**G**–**I**) After 24 h of incubation with 200 nM Ang II with or without 20 μM GW4869, HUVECs were co-cultured with HASMCs for another 24 h. Relative miR-92a levels in EC-derived EVs (**G**) and HASMCs (**H**). The relative expression of indicated genes in HASMCs in the three groups (**I**). (**J**) Naïve VSMCs were treated with EVs from conditioned media of Ang II- or PBS-treated HUVECs for 24 h. (**K**, **L**) qPCR analysis of miR-92a mRNA level in HUVECs and VSMCs. (**M**) The relative expression of indicated genes in VSMCs treated with EC-derived EVs from Ang II or Ctrl groups. Data are mean ± SEM from 6 independent experiments (**B**–**I**) and 3 independent experiments (**K**–**M**). Normally distributed data were analyzed by the two-tailed Student *t* test (**C**, smtn, calponin, and FN in **E**, and **K**–**M**) and non-normally distributed data were analyzed by the Mann–Whitney U test (**B**, **D** and α-SMA, OPN, and Thbs in **E**) between 2 indicated groups. Normally distributed data (**G**–**I**) were analyzed by the two-way ANOVA between multiple groups. *p < 0.05, **p < 0.01, ***p < 0.001, ****p < 0.001. The schematic diagram (**A**, **J**) were generated by Microsoft PowerPoint & Visio 2019 MSO (16.0.14326.20384), 64 bits, http://www.microsoft.com.

HUVECs were treated with Ang II or PBS for 24 h before co-culture with VSMCs (Fig. 4A). Level of miR-92a was elevated in ECs treated with Ang II and in EVs isolated from conditioned media of Ang II-treated ECs (Fig. 4B,C). Furthermore, miR-92a level was elevated in VSMCs co-cultured with Ang II-treated ECs, as compared with phosphate buffered saline (PBS)-treated ECs (Fig. 4D). We then measured mRNA levels of genes related to the contractile and proliferative phenotype. The mRNA levels of contractile markers (α-SMA, smoothelin, and calponin) were decreased, but those of proliferative markers (fibronectin, osteopontin, and thrombospondin) were increased in VSMCs co-cultured with Ang II-treated ECs (Fig. 4E). Given that miR-92a level is significantly increased in carotid arteries after intima injury^13^, we analyzed RNA-seq data (GSE164050) from carotid arteries. Consistent with our results, marker genes of the contractile phenotype of VSMCs were downregulated, and those of the proliferative phenotype were upregulated in carotid arteries (Fig. 4F). We next investigated that EC-derived EVs stimulated by Ang II are responsible for the contractile-to-synthetic phenotype change of VSMCs. HUVECs were incubated with Ang II with 20 μM GW4869 for 24 h to block the formation of EVs or without, prior to ECs co-cultured with VSMCs. As shown in Fig. 4G–I, GW4869 treatment mitigated the contractile-to-synthetic phenotype change of VSMCs. We further treated naïve VSMCs with EVs isolated from conditioned media of Ang II-treated ECs (Fig. 4J). Level of miR-92a was increased in ECs and VSMCs with EVs incubation (Fig. 4K,L). Also, mRNA levels of fibronectin, osteopontin, and thrombospondin were increased, but levels of α-SMA, smoothelin, and calponin mRNA were decreased in VSMCs incubated with EC-derived EVs (Fig. 4M). Collectively, results in Figs. 3 and 4 suggest that Ang II-induced miR-92a level in ECs could translocate to neighboring VSMCs via EVs, which leads to the contractile-to-synthetic phenotype change of VSMCs.

### LNA-miR-92a reduces hypertension susceptibility

**Figure 5:**
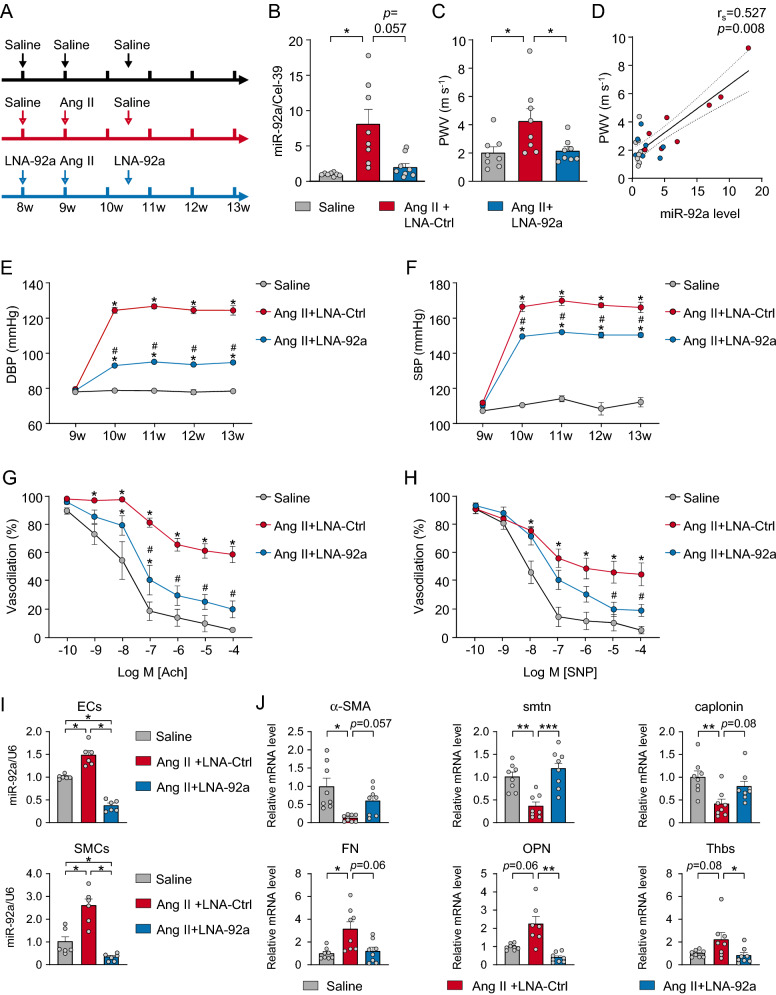
LNA-miR-92a reduces hypertension susceptibility. (**A**) Experimental design of mouse study. Ang II was released by osmotic minipumps at 1 μg/kg/min for 4 weeks. One week before Ang II infusion, LNA-control (LNA-Ctrl) or LNA-miR-92a was delivered at 16 mg/kg body weight via tail-vein injection, followed by a second dose of LNA-Ctrl or LNA-miR-92a delivery 10 days after the Ang II minipump implantation. The animals were sacrificed at the end of 4-week post-minipump implantation. (**B**) miR-92a level was measured in CD144^+^-EVs isolated from serum (n = 8). (**C**) Left carotid artery PWV (n = 8). (**D**) Correlation between the level of serum miR-92a and PWV. (**E**, **F**) Ambulatory SBP and DBP (n = 8). (**G**, **H**) Thoracic aortic rings were isolated from three groups of mice (n = 5). Representative traces of the ACh- (**G**) and SNP-induced (**H**) relaxation of Phe-precontracted rings. The dots represented cumulative addition of increasing doses of ACh and SNP (5 × 10^–10^ to 10^–4^ M). (**I**) qPCR analysis of miR-92a level in aortic ECs and SMCs from mice (n = 6). (**J**) qPCR analysis of indicated genes in aortas from mice (n = 8). Data are mean ± SEM. Normally distributed data were analyzed by the one-way ANOVA (**B**, **C**, ECs in I, smtn, calponin, FN, OPN and Thbs in **J**) between multiple groups. Normally distributed data (E–H) were analyzed by the two-way ANOVA between multiple groups. Non-normally distributed data were analyzed by the Kruskal–Wallis test (SMCs in I, α-SMA in **J**) between multiple groups. *p < 0.05 vs. Saline, ^#^*p* < 0.05 vs. Ang II + LNA-Ctrl. *p < 0.05, **p < 0.01, ***p < 0.001. r_s_, Spearman correlation coefficient.

For translational relevance, we tested the efficacy of exogenously delivered miR-92a in suppressing Ang II-induced hypertension and arterial stiffness. We administered LNA-miR-92a to C57BL/6 mice via tail vein injection (Fig. 5A). LNA-miR-92a administration significantly suppressed the level of miR-92a in circulating CD144^+^-EVs and mitigated the Ang II-elevated PWV as compared with control LNA (LNA-Ctrl) (Fig. 5B,C). Moreover, the level of miR-92a in circulating CD144^+^-EVs was positively correlated with PWV in the three groups of mice (Fig. 5D). Consistently, LNA-miR-92a administration reduced the Ang II-elevated SBP and DBP (Fig. 5E,F). With vasorelaxation assays of isolated aortic segments, we assessed the functional outcomes of LNA-miR-92a at the tissue level. The eNOS-dependent vasodilation induced by acetylcholine (ACh) and the endothelium-independent vasodilation induced by sodium nitroprusside (SNP) were impaired in aortas from mice receiving Ang II (Fig. 5G,H). LNA-miR-92a administration reversed both the EC-dependent and -independent vasodilation.

To further elucidate the role of miR-92a in VSMC phenotype transition in this mouse model, we assessed transcripts in the mouse aorta that are related to contractile versus proliferative phenotypes. As compared with LNA-Ctrl administration, LNA-miR-92a administration significantly reduced the Ang II–elevated miR-92a levels in aortic ECs and SMCs (Fig. 5I). Consistently, mRNA levels of α-SMA, smoothelin, and calponin were decreased, whereas those of fibronectin, osteopontin, and thrombospondin were increased in aortas of mice receiving Ang II and LNA-miR-92a (Fig. 5J). Thus, the exogenously administered LNA-miR-92a was effective to mitigate arterial stiffness associated with Ang II-induced hypertension in mice. To demonstrate that miR-92a in CD144^+^-EVs is crucial for the phenotypic changes of VSMCs in vivo, we also isolated serum CD144^+^-EVs from mice treated with Ang II or saline, and then injected these EVs into the wild-type mice. As shown in Supplementary Fig. S3, the serum and VSMC miR-92a levels in mice administered CD144^+^-EVs isolated from Ang II-treated mice increased significantly. Consistently, the VSMC contractile markers in mice receiving these CD144^+^-EVs significantly decreased, while the proliferative markers increased. These data indicate that the CD144^+^-EVs associated miR-92a would contribute, at least in part, to the vascular dysfunction and VSMC phenotype transition in Ang II-administered mice.

## Discussion

**Figure 6:**
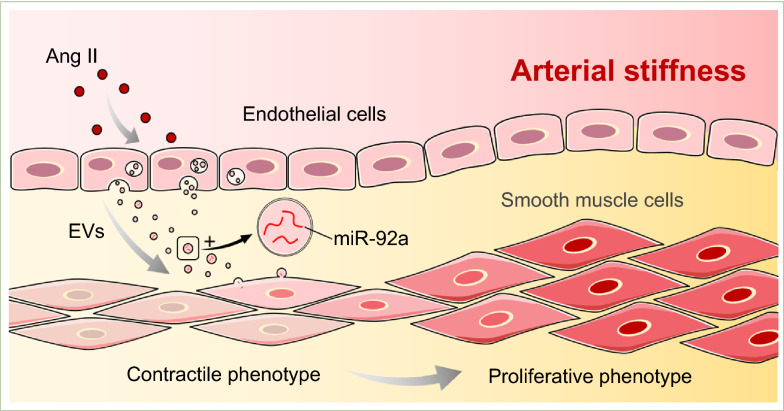
Schematic illustration of EC miR-92a regulation of VSMCs plasticity under Ang II stimulation. Ang II-induced EC dysfunction can result in an extracellular transfer of EC-miR-92a to the neighboring VSMCs via EVs to modulate the contractile-to-proliferative phenotypic change of VSMCs, which led to arterial stiffness. The schematic diagram was generated by Microsoft PowerPoint & Visio 2019 MSO (16.0.14326.20384), 64 bits, http://www.microsoft.com.

Arterial stiffness is considered a complication of hypertension caused by the long-term adverse effects of elevated blood pressure, among other risk factors. Reciprocally, vascular stiffness is a pathological culprit of hypertension^2^. At the molecular level, arterial stiffness is affected by VSMC tone and EC signaling resulting from aging, vascular growth factors, calcification, and activation of innate and adaptive immunity^2,17^. In this study, we found that a cross-talk between dysfunctional ECs and VSMCs can promote arterial stiffness. Specifically, elevated abundance of extracellular miR-92a is transported from impaired ECs to VSMCs, which promotes vascular stiffness (Fig. 6).

Highly expressed in ECs, miR‐92a induces EC dysfunction and also increases VSMC proliferation, migration, and apoptosis^18–23^. MiR-92a can mediate cell-to-cell communication within the vasculature via EVs. Upon vascular damage, activated platelets adhere to the intima, then secrete platelet-derived EVs. These miR-92a-containing EVs, when transported to VSMCs, can induce Col8a1, which augments vascular stiffness^13^. Our results showed that Ang II increased miR-92a level in ECs, which resulted in EC dysfunction. Thus, EC–VSMC communication involving miR‐92a might also contributes to the phenotype changes of VSMCs in our experimental conditions. To test this hypothesis, we co-cultured VSMCs with dysfunctional ECs. Results in Figs. 2, 3 and 4 show that impaired ECs with high miR-92a expression upregulated genes promoting proliferation in the co-cultured VSMCs. Thus, the contractile-to-proliferative phenotype change of VSMCs can be induced by miR-92a secreted from ECs. In line with this result, miR-92a level was markedly increased in EVs secreted from Ang II-treated ECs and in co-cultured VSMCs (Fig. 4). Analysis of RNA-seq datasets (GSE164050) revealed that the transcriptomes of injured carotid arteries contained high miR-92a level transported by activated platelets. This in silico analysis showed that the expression of genes related to the contractile phenotype was decreased whereas that related to the proliferative phenotype was increased in the injured carotid artery as compared with controls.

The miR-92a-induced EC dysfunction would be contributed by miR-92a-targeted KLF2, KLF4, and SIRT1^14,18,21^. Whether endothelial KLF2, KLF4 or SIRT1 can transport to VSMCs is unknown. However, VSMC-specific SIRT1 ablation worsens the oxidative and inflammatory responses to Ang II^24^. Thus, besides miR-92a, the proteins targeted by miR-92a may also participate in the EC-VSMC crosstalk. Several studies have reported that Ang II induces the proliferative phenotypic change of VSMCs^25,26^. We also detected the level of miR-92a in VSMCs treated with Ang II, and found that Ang II increased the expression of miR-92a in VSMCs (Supplementary Fig. S2). Thus, we cannot rule out the possibility that Ang II directly regulates contractile-to-synthetic phenotype gene expression changes in VSMCs through changes in SMC miR-92a level.

Macrophages play a critical role in vascular inflammation and remodeling by releasing proinflammatory cytokines and growth factors that act on neighboring ECs and VSMCs^27–29^. Of note, EC miR-92a can be also transported to macrophages via EVs. Ample evidence shows that circulating miR-92a level is associated with cardiovascular diseases^14–16,30^. Reducing miR-92a level in animal models attenuates EC dysfunction and atherosclerosis^14,19^. Circulating miR-92a level was found correlated with PWV in hypertensive patients^16^. In the current study, serum miR-92a level was inversely correlated with PWV in humans. In the Ang II-induced mouse hypertension model, exogenously administered LNA-miR-92a could alleviate Ang II-induced hypertension and arterial stiffness (Fig. 5). LNA-miR-92a may have efficacy in ECs, VSMCs, and macrophages, to collectively ameliorate vascular stiffness. In summary, our study demonstrated that (1) circulating miR-92a level was increased in both hypertensive patients and Ang II-induced hypertensive mice in comparison with healthy controls; (2) circulating miR-92a level was positively correlated with PWV, SBP, and DBP but inversely with NO level in humans; (3) Ang II caused EC dysfunction via EC-derived miR-92a; (4) miR-92a-enriched EVs secreted by dysfunctional ECs played a critical role in modulating the VSMC phenotype transition; and (5) exogenously administered LNA-miR-92a alleviated Ang II-induced hypertension and arterial stiffness in mice. Circulating miR-92a level could be an important biomarker and a potential therapeutic target for hypertension and arterial stiffness.

## Methods

### Human participants and serum samples

A total of 44 patients were prospectively enrolled from October to December 2017 at Xi'an GaoXin Hospital, Xi’an, China. Patients included had a first diagnosis of hypertension according to recommendations of the 2017 Chinese Guidelines for the diagnosis and management of arterial hypertension (i.e., SBP ≥ 140 mmHg and/or DBP ≥ 90 mmHg at rest). Exclusion criteria included receiving relevant medications for hypertension (i.e., lipid-lowering drugs, anti-platelet or anti-hypertensive drugs), hyperlipidemia, diabetes, hyperhomocysteinemia, coronary heart disease, cerebral infarction, stroke, chronic obstructive pulmonary disease, liver and kidney failure, severe infection, and malignant tumor. A group of 21 age- and sex-matched healthy controls were recruited from Xi'an GaoXin Hospital as well. The study was approved by Institutional Ethics Committee of Xi’an GaoXin Hospital, and written informed consent was obtained from all participants. Blood samples were taken from patients or healthy controls after 12-h overnight fasting. Serum was collected by centrifugating whole blood at 1500*g*$\times$ for 10 min and quickly frozen at − 80 °C for storage until use. Serum NO level was assessed by a NO assay kit (Beyotime) according to the manufacturer’s instructions. Serum ET-1 level was measured by ELISA (SHHY, China), according to the manufacturer’s instructions. The experiments were approved by the Ethics Committee of Xi’an Jiaotong University and were carried out in accordance with relevant guidelines and regulations.

### Brachial-ankle PWV (baPWV) measurement

All participants were asked to rest for at least 10 min in the supine position before baPWV measurement. baPWV was measured with an automatic waveform analyzer (VP-1000, Colin, Japan), which automatically records pulse waves of the brachial and posterior tibial arteries with automated oscillometric sensors. After obtaining 5 min of bilateral baPWV values, the average value was used for further analysis.

### Animal experiments

All animal experiments were approved by the Institutional Animal Ethics Committee of Xi’an Jiaotong University and were carried out in accordance with relevant guidelines and regulations. All mice (C57BL/6J) were kept on a 12-h light/dark cycle and fed a chow diet ad libitum at room temperature. For Ang II-induced hypertension, mice at 9 weeks old were subcutaneous infused with Ang II at 1 μg/kg/min for 28 days by Osmotic minipumps (model 2004, Alzet). One week before Ang II infusion, mice received LNA-Ctrl or LNA-miR-92a at 16 mg/kg body weight by tail-vein injection (Fig. 5A). The second dose of LNA-miR-92a was given 10 days after the minipump implantation. Mice were euthanized by intraperitoneal injection of 200 μL of 2% pentobarbital sodium at the end of day 28 after minipump implantation. LNAs were designed and synthesized by GenePharma Co. Blood pressure was measured by the tail-cuff method (BP-2000, Visitech Systems, Inc.) at Xi’an Jiaotong University. We confirm that the study was carried out in compliance with the ARRIVE guidelines.

### Mouse left carotid artery PWV (caPWV) measurement

Mice were anesthetized by inhaled isoflurane via a facemask, then underwent transthoracic echocardiography with a Vevo 2100 instrument (VisualSonics Inc., Toronto, ON, Canada) equipped with a MS-700 transducer (50 MHz). On the same image plane, the left caPWV was measured: (1) times T1 and T2 were measured in the proximal internal or distal internal carotid artery, respectively; (2) distance (D) in the carotid artery between the two sample volume positions was measured; (3) caPWV was calculated as PWV (cm/s) = D/(T1 − T2).

### Vascular vasodilation

After mice were sacrificed, thoracic aortas were removed and cleaned in oxygenated ice-cold Na^+^-Krebs buffer. Thoracic aortas were cut into 2-mm aortic rings and transferred to fresh Krebs solution for functional studies with the Myograph system (Danish Myo Technology, Aarhus, Denmark) to measure vasorelaxation. All rings were stretched to an optimal baseline tension (3 mN) and equilibrated for 1 h before contraction by phenylephrine (Phe, 10 μM). The changes in isometric tension were recorded by using the PowerLab Data Acquisition System (Harvard Apparatus, Holliston, MA, USA). Phenylephrine (Phe), acetylcholine (ACh), and sodium nitroprusside (SNP) were from Sigma-Aldrich (St. Louis, MO, USA) and dissolved in sterile ddH_2_O.

### Cell culture and EC-SMC co-culture

Human artery smooth muscle cells (HASMCs) were obtained from Sciencell Co. and cultured in smooth muscle cell medium (SMCM; Sciencell) on poly-l-lysine–coated cell culture flasks. Primary cultures of cells at passages 4–8 were used in all experiments. Human umbilical vein cells (HUVECs) were cultured in M199 medium (Sigma-Aldrich) supplemented with 15% fetal bovine serum, 5 ng/ml recombinant human fibroblast growth factor, 90 µg/ml heparin, 20 mM HEPES (pH 7.4), and 100 U/ml penicillin–streptomycin. All cell cultures were maintained at 37 °C in 95% air and 5% CO_2_. The EC-SMC coculture was conducted with transwell inserts at 0.4-μm pore size (Corning) in 6-well plates. HUVECs were seeded onto the upper compartment of the insert and treated with 200 nM Ang II (MedChemExpress, MCE) or phosphate buffered saline (PBS) for 24 h before co-culture with HASMCs. HASMCs were seeded at the bottom of the 6-well plate and cocultured with pre-treated HUVECs for 24 h (Fig. 4A).

### Extracellular vesicle (EV) isolation, analysis, and labeling

CD144-enriched EVs were isolated with procedures described previously^15^ with minor modification. Cells, dead cells, and cell debris were removed from the conditioned media by centrifugation at 300*g*$\times$ for 5 min, 2000*g*$\times$ for 15 min and 10,000*g*$\times$ for 30 min, respectively. After centrifugation, the supernatant was ultracentrifuged at 120,000*g*$\times$ at 4 °C for 70 min to pellet the small vesicles (Beckman Optima L-100XP, SW40Ti). The serum was collected and diluted with PBS in a 1:1 ratio, and then ultracentrifuged at 200,000*g*$\times$ at 4 °C for 2 h. The pellet was washed with PBS and filtrated through a 0.22-μm filter. Then, the pellet was immunoblotted with anti-CD144 antibody (Santa Cruz Biotechnology) and incubated with Dynabeads (Invitrogen). Total RNA from CD144-enriched EVs was isolated with TRIzol and with Cel-miR-39 at 2 nM added as a spike-in control. For electron microscopy analysis, the EVs were fixed in 4% paraformaldehyde at 4 °C for 1 h, and then placed on a formvar-coated grid and negatively stained with 2% (w/v) uranyl acetate. Sections were observed using transmission electron microscopy (HITACHI, HT7800). For nanoparticle tracking analysis (NTA) of EVs: Isolated exosome samples were appropriately diluted using 1$\times$ PBS buffer (Biological Industries, Israel) to measure the particle size and concentration. NTA measurement was recorded and analyzed at 11 positions. Particle sizes of EVs were analyzed by nanoparticle tracking analysis using a ZetaView PMX110 (Particle Metrix, Meerbusch, Germany) and the corresponding software ZetaView 8.04.02 SP2. For EVs uptake: A total of 2 × 10^5^ HASMCs were incubated with 2 μl PKH63 (Sigma-Aldrich)-labeled EC-derived EVs in 24-well plates for 24 h at 37 °C in 95% air and 5% CO_2_. The cells were fixed in 4% paraformaldehyde for 15 min and washed twice with PBS, and then incubated with antibodies against α-SMA, followed by secondary antibody. The samples were counterstained with DAPI, then mounted with fluorescent mounting medium. An Olympus IX81 fluorescence microscope was used to acquire fluorescence images.

### Western blot analysis and RT-qPCR

For western blot analysis, cells were lysed in RIPA buffer supplemented with protease inhibitors (Thermo Scientific). Lysates were loaded by SDS-PAGE and immunoblotted with antibodies as indicated. For quantitative real-time PCR, total RNA and miRNA were extracted by using TRIzol reagent (Invitrogen). For serum samples, RNA was isolated by using TRIzol from 200 µl serum. Cel-miR-39 at 2 nM was added as a spike-in control. The mRNA or miR expression was determined by using SYBR Green (Bio-Rad) or TaqMan probe-participated qPCR; β-actin and U6 were internal controls for normalization of mRNA and miR expression, respectively. The sequences for qPCR primers are in Table 2. The primary antibodies used were anti-KLF4 (Cell Signaling Technology, 12173S), anti-KLF2 (Abcam, ab17008), anti-eNOS (BD Transduction Laboratories, 610296) and anti-ET-1 (Abcam, ab2786). Secondary antibodies were anti-mouse (Jackson, 515-035-003) and anti-rabbit antibodies (Jackson, 111-035045).

**Table 2:** Primers used for qPCR.

Gene	Forward 5′–3′	Reverse 5′–3′
**For human**		
β-Actin	CATGTACGTTGCTATCCAGGC	CTCCTTAATGTCACGCACGAT
KLF2	TTCGGTCTCTTCGACGACG	TGCGAACTCTTGGTGTAGGTC
KLF4	CCCACATGAAGCGACTTCCC	CAGGTCCAGGAGATCGTTGAA
eNOS	TGATGGCGAAGCGAGTGAAG	ACTCATCCATACACAGGACCC
ET-1	GGGCTGAAGACATTATGGAG	CGAAGGTCTGTCACCAATGT
α-SMA	AAAAGACAGCTACGTGGGTGA	GCCATGTTCTATCGGGTACTTC
Smtn	CCCTGGCATCCAAGCGTTT	CTCCACATCGTTCATGGACTC
Calponin	CTGTCAGCCGAGGTTAAGAAC	GAGGCCGTCCATGAAGTTGTT
FN	CGGTGGCTGTCAGTCAAAG	AAACCTCGGCTTCCTCCATAA
OPN	CTCCATTGACTCGAACGACTC	CAGGTCTGCGAAACTTCTTAGAT
Thbs	AGACTCCGCATCGCAAAGG	TCACCACGTTGTTGTCAAGGG
**For mouse**		
β-Actin	GGCTGTATTCCCCTCCATCG	CCAGTTGGTAACAATGCCATGT
α-SMA	GTCCCAGACATCAGGGAGTAA	TCGGATACTTCAGCGTCAGGA
Smtn	GCCCTCAGATACCTTGGACTC	GGCAGGATTTCGTTTCAGACG
Calponin	TCTGCACATTTTAACCGAGGTC	GCCAGCTTGTTCTTTACTTCAGC
FN	ATGTGGACCCCTCCTGATAGT	GCCCAGTGATTTCAGCAAAGG
OPN	CACTCCAATCGTCCCTACAGT	CTGGAAACTCCTAGACTTTGACC
Thbs	GTGAGGTTTGTCTTTGGAACCA	GTTGTTGTCAAGGGTAAGAAGGA

### Statistical analysis

Analyses were performed with GraphPad Prism 9. For normally distributed data, the two-tailed Student *t* test was used to compare two groups and ANOVA with Bonferroni post-hoc test for multiple groups. For non-normally distributed data, the Mann–Whitney U test was used to compare two groups and Kruskal–Wallis test for multiple groups. Correlational analyses involved using Spearman correlation. Data are expressed as mean ± SEM and *p < 0.05 was considered statistically significant.

### Consent for publication

The authors agree for publication.

## Supplementary Information

Supplementary Figures.

## Data Availability

The datasets generated during and/or analyzed during the current study are available from the corresponding author on request.
