# Supplementary material for: Endothelial-derived extracellular microRNA-92a promotes arterial stiffness by regulating phenotype changes of vascular smooth muscle cells
Source: Sci Rep. 2022 Jan 10;12:344. doi: 10.1038/s41598-021-04341-1 (PMC8748448; doi:10.1038/s41598-021-04341-1)
Supplement: Supplementary file 1 — Supplementary Figures. [file 41598_2021_4341_MOESM1_ESM.docx]

**SUPPLEMENTARY FILE**

**Endothelial**-**derived extracellular microRNA**-**92a promotes arterial stiffness by regulating phenotype changes of vascular smooth muscle cells**

Chen Wang, BS^1,3†^, Haoyu Wu, MS^1†^, Yuanming Xing, BS^1,3^, Yulan Ye, BS^5^, Fangzhou He, BS^3^, Qian Yin, MD^1,3^, Yujin Li, BS^6^, Fenqing Shang, MD^2,3^*, John Y-J Shyy, PhD^4^, Zu-Yi Yuan, MD^1^*.

^1^Department of Cardiology, First Affiliated Hospital of Xi’an Jiaotong University, Xi'an, China. ^2^Translational Medicine Centre, Xi’an Chest Hospital, Xi'an, China. ^3^Cardiovascular Research Center, School of Basic Medical Sciences, Xi’an Jiaotong University Health Science Center, Xi'an, China. ^4^Division of Cardiology, Department of Medicine, University of California, San Diego, La Jolla, CA. ^5^Department of Cardiology, Xi’an GaoXin Hospital, Xi'an, China. ^6^Department of Life Sciences and Medicine, Northwestern University, Xi'an, China.

^†^ Chen Wang and Haoyu Wu are co-first authors.

* Zu-Yi Yuan and Fenqing Shang are corresponding authors.

**Address for Correspondence:**

Zu-Yi Yuan, MD, PhD,

E-mail: [zuyiyuan@mail.xjtu.edu.cn](mailto:zuyiyuan@mail.xjtu.edu.cn)

Fenqing Shang, MD, PhD,

E-mail: shangfenqing@163.com


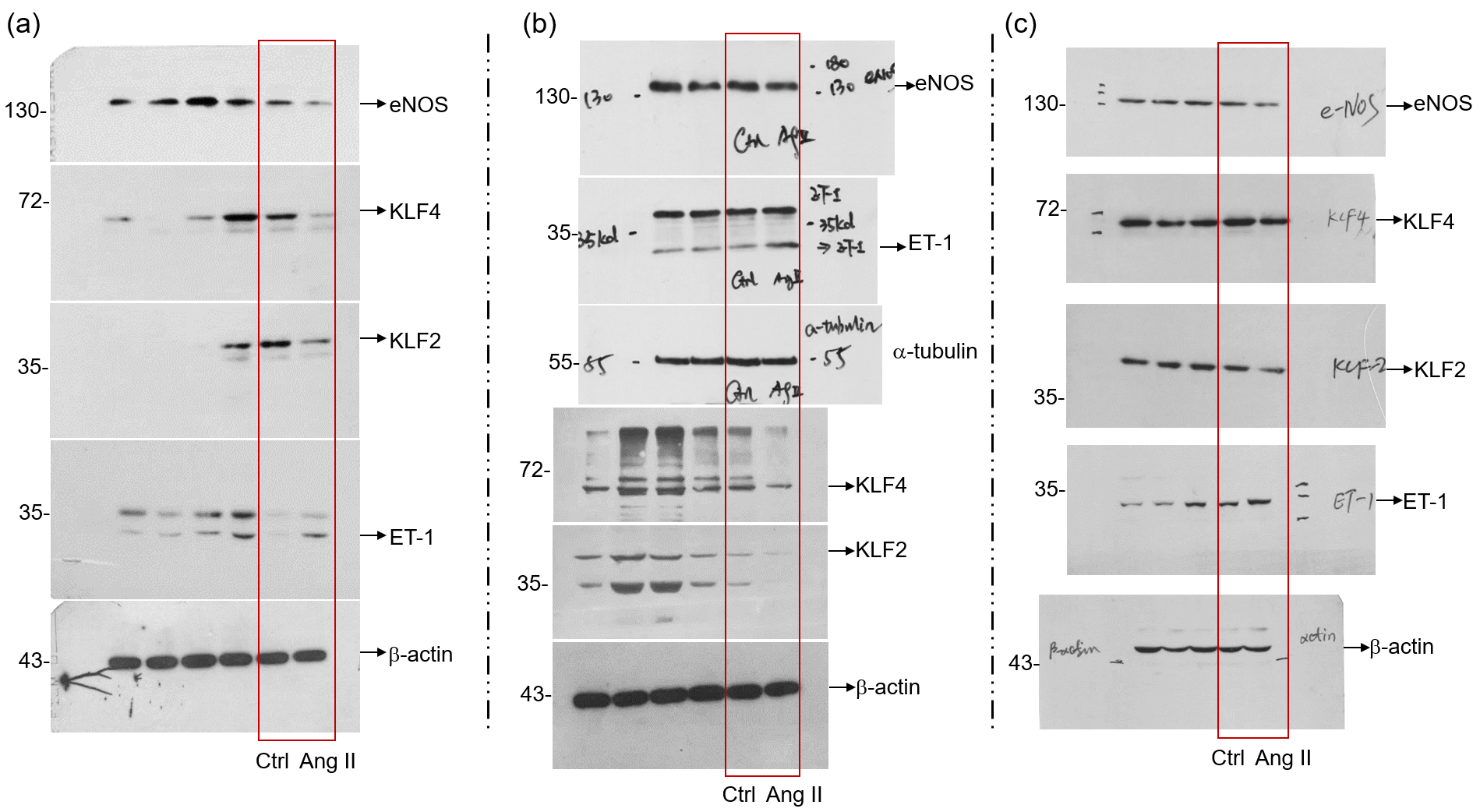


Supplementary Fig. S1. Original Western blot gels. **(a-c)** HUVECs were treated with Ang II (200 nM) or PBS (Ctrl) for 24 hr. Full gel pictures showing the bands that correspond to the expression of eNOS, KLF4, KLF2, and ET-1, with β-actin/α-tubulin as a loading control. The experiment was repeated three times.


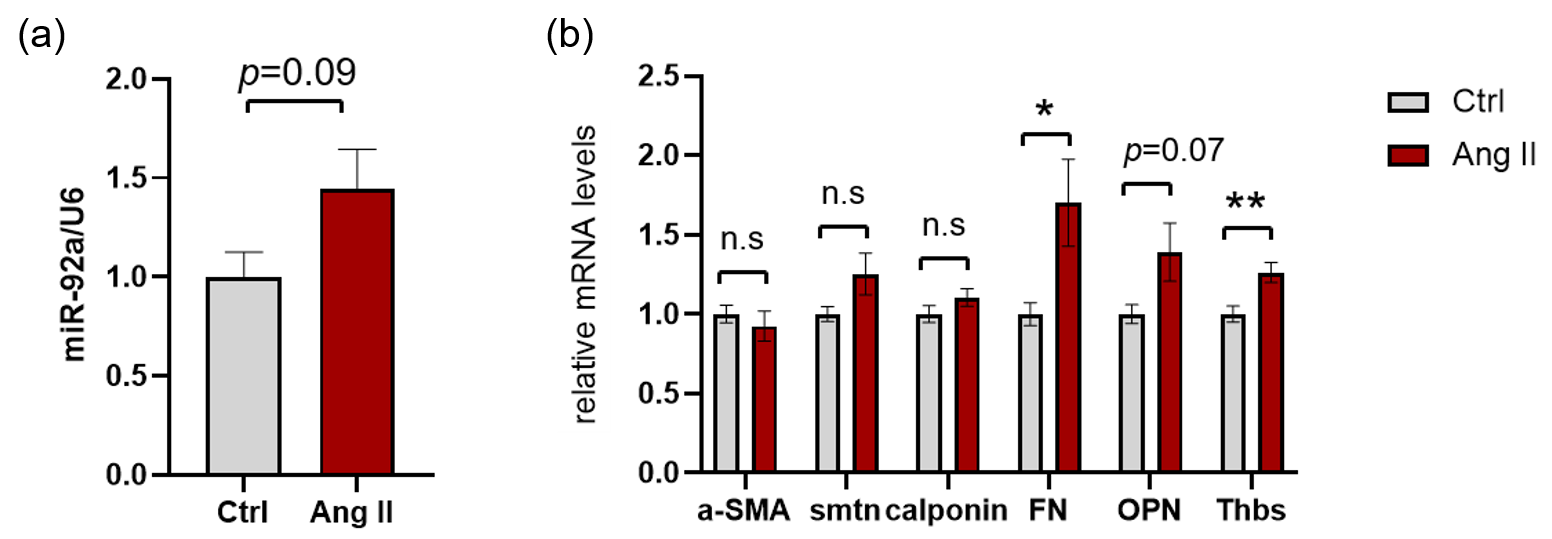


Supplementary Fig. S2. HASMCs were treated with 200 nM Ang II or PBS (Ctrl) for 24 hr. **(a-b)** qPCR analysis of miR-92a level (a) and indicated genes (b) in HASMCs. Data are mean ± SEM from 6 independent experiments (a-b). Normally distributed data were analyzed by the two-tailed Student *t* test (a, α-SMA, smtn, calponin, OPN, and Thbs in b) and non-normally distributed data were analyzed by the Mann-Whitney U test (FN in b). **p*<0.05, ***p*<0.01.


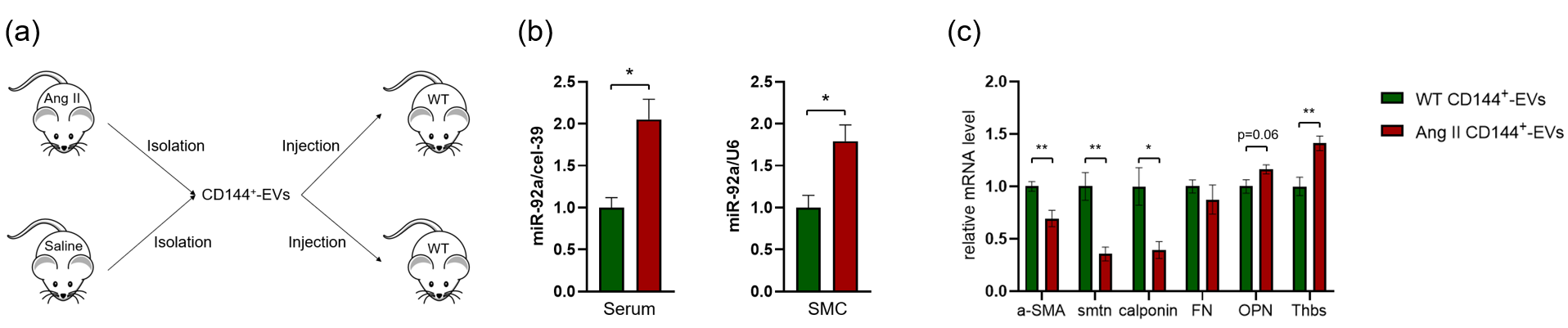


Supplementary Fig. S3. **(a)** Experimental design of mouse study. Ang II was released by osmotic minipumps at 1 μg/kg/min for 2 weeks. Isolated serum CD144^+^-EVs from mice treated with Ang II or saline, and then injected these EVs into the wild-type mice. **(b)** qPCR analysis of miR-92a levels in serum and aortic SMCs from mice. **(c)** qPCR analysis of indicated genes in aortic SMCs from mice. Data are mean ± SEM from 6 independent experiments. Normally distributed data were analyzed by the two-tailed Student *t* test (b, α-SMA, smtn, calponin, OPN, and Thbs in c) and non-normally distributed data were analyzed by the Mann-Whitney U test (FN in c). **p*<0.05, ***p*<0.01.
